# *In vitro* release studies of furosemide reference tablets: influence of agitation rate, USP apparatus, and dissolution media

**DOI:** 10.5599/admet.801

**Published:** 2020-06-29

**Authors:** Raúl Medina-López, Sergio Guillén-Moedano, Marcela Hurtado

**Affiliations:** Departamento Sistemas Biológicos, Universidad Autónoma Metropolitana-Xochimilco, Mexico City, Mexico

**Keywords:** Flow-through cell method, Furosemide, Lasix® drug product, USP basket and paddle apparatus

## Abstract

Furosemide is a diuretic drug widely used in chronic renal failure. The drug has low solubility and permeability, which cause clinical problems. Studying the in vitro release performance elucidates the rate and extent of drug dissolved from dosage forms under different conditions. Furosemide reference tablets were tested using USP Apparatuses 1 and 2 as well as the flow-through cell method (USP Apparatus 4), a dissolution apparatus that simulates the human gastrointestinal tract better than the other methods. Dissolution profiles were created with USP Apparatuses 1 and 2 at 25, 50, and 75 rpm and 900 mL of 0.1 M hydrochloric acid, acetate buffer (pH 4.5), and phosphate buffer (pH 6.8). USP Apparatus 4 with a laminar flow of 16 mL/min and 22.6 mm cells was used. Drug dissolution was quantified at 274 nm for 60 min. Mean dissolution time, dissolution efficiency, time to 50% dissolution, and time to 80% dissolution data were used to compare dissolution profiles. Additionally, zero-order, first-order, Higuchi, Hixson-Crowell, Makoid-Banakar, and Weibull models were used to adjust furosemide dissolution data. Between USP Apparatus 1 and 2, significant differences were observed in almost all parameters at 50 and 75 rpm (p < 0.05). A similar dissolution profile (f_2_ > 50) with a pharmacopoeial dissolution method (USP Apparatus 2 at 50 rpm and 900 mL of phosphate buffer pH 5.8) and USP Apparatus 4 (laminar flow of 16 mL/min, 22.6 mm cells, and pH 6.8) was observed. The Weibull function was the best mathematical model to describe the in vitro release performance of furosemide in the three USP dissolution apparatuses. These results could be used to manufacture better furosemide dosage forms and decrease the negative clinical impact of current furosemide formulations.

## Introduction

**Figure 1.**
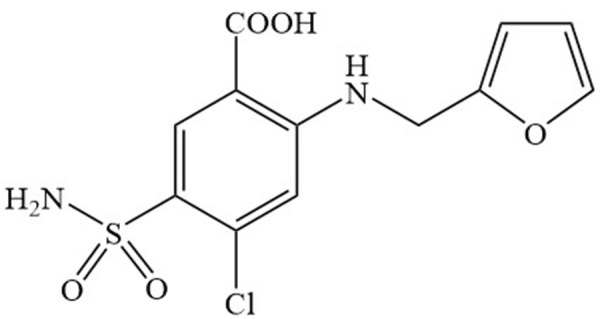
Chemical structure of furosemide.

Furosemide is a diuretic drug widely used in the treatment of oedematous states associated with cardiac, chronic renal failure, hypertension, congestive heart failure, and cirrhosis [1]. Furosemide is a weak acid (p*K*a = 3.8) with low solubility and permeability [2]. According to the Biopharmaceutical Classification System, drugs with these characteristics belong to class IV [3]. The chemical structure of furosemide is shown in Figure 1.

Factors that affect drug bioavailability related to the pharmaceutical dosage form and manufacturing process have been described [4, 5]. Techniques and strategies for the development of class IV drug formulations have also been discussed [6]. This information can support the design of better oral dosage forms of furosemide than those currently available. The oral bioavailability of furosemide has been reported to be 37–51% [7] and 60–70%, with a variable and erratic absorption [8]. For the reference product Lasix®, an absolute bioavailability of 56% has been observed [9]. This limited bioavailability could be associated with significant differences in the dissolution behaviour of furosemide commercial formulations shown by several authors. Kaojarern *et al*. [10] reported an *in vitro* dissolution study of 13 brands of furosemide tablets (40 mg), of which only four fulfil the Q pharmacopoeial specification. Stüber *et al*. [11] studied the bioavailability of four furosemide drug products, of which three formulations have bioavailabilities of 81–83% and lower *in vitro* dissolution performance than the reference in each of following conditions: pH 7.8/paddle at 25 rpm, pH 7.8/paddle at 50 rpm, pH 5.3/paddle at 50 rpm, and flow-through cell (100 mL/h)/pH 7.8. The difference in *in vitro* dissolution is more pronounced at pH 5.3/paddle at 50 rpm. Currently, the official dissolution test for furosemide tablets is the USP Apparatus 2 at 50 rpm with 900 mL of phosphate buffer pH 5.8 and no less than 80% should be dissolved at 60 min [12]. A biowaiver monograph has been reported to waiver the *in vivo* bioequivalence of furosemide solid oral dosage forms by *in vitro* dissolution studies; however, given the available data, Granero *et al*. [3] concluded that a biowaiver procedure for this drug cannot be justified.

Low solubility and permeability are problematic characteristics of class IV drugs; thus, the determination of *in vitro* release performance using different agitation rates, dissolution media, and dissolution apparatuses provides important information for improving the manufacture and evaluation of generic formulations. Despite the wide use of USP basket and paddle apparatuses (USP Apparatus 1 and 2, respectively) to monitor the physical quality of tablets and capsules, several investigations about the hydrodynamic environment that surrounds oral formulations have reported that these USP apparatuses do not adequately reproduce the natural environment of the gastrointestinal tract [13-15]; thus, it is necessary to document the *in vitro* release performance of poorly soluble drugs under different conditions to establish, in the best possible way, the environment in which the solid dosage forms will be within the gastrointestinal tract. Further, alternative apparatuses must be developed to achieve this goal. The flow-through cell method (USP Apparatus 4) has been introduced as a dissolution apparatus to elucidate the rate and extent of the dissolution of drugs with low solubility under sink conditions [16]. USP Apparatus 4 is more reliable, reproducible, and discriminative than the other methods [17], and it generates a hydrodynamic environment similar to that inside the gastrointestinal tract [18]. Furthermore, an *in vitro*/*in vivo* correlation (IVIVC) has been established between the *in vitro* data generated with this apparatus and *in vivo* parameters [19, 20].

The aim of this study was to evaluate the *in vitro* release performance of furosemide reference tablets in the hydrodynamic environments generated by different USP apparatuses and dissolution media of physiological relevance to identify the rate and extent of furosemide dissolution under these conditions. This information will reflect the characteristics of the reference drug product that can be considered in the preparation of a generic formulation. Lasix® tablets were tested with 0.1 M hydrochloric acid, acetate buffer (pH 4.5), and phosphate buffer (pH 6.8) using USP Apparatuses 1 and 2 (at different agitation rates) and USP Apparatus 4.

## Experimental

### Materials

Lasix® furosemide tablets (Sanofi-aventis de Mexico, S.A. de C.V., Mexico City, Mexico) were used. Mexican health authorities have designated this formulation as a reference product to be used in dissolution and bioequivalence studies [21].

### Reagents

Hydrochloric acid, sodium acetate, acetic acid, and phosphate salts were purchased from J.T. Baker-Mexico (Xalostoc, Mexico). Furosemide reference standard was purchased from Sigma-Aldrich Co. (St. Louis, MO, USA). All samples were filtered through 0.45 μm nitrocellulose filters (Millipore, Ireland). Standard solutions were prepared by serial dilutions of the stock solutions of furosemide (1 mg/mL) to achieve concentrations of 1.25–20 μg/mL. The dissolution media comprised 0.1 N hydrochloric acid, acetate buffer (pH 4.5), and phosphate buffer (pH 6.8).

### Content uniformity and assay

Content uniformity and assay tests were performed with the drug product according to the procedures described in the USP [12].

### USP basket and paddle apparatus

Dissolution profiles of furosemide were obtained using USP Apparatuses 1 and 2 (Model AT-7 Smart, Sotax, Basel, Switzerland) with 25, 50, and 75 rpm agitation rates. Additionally, pharmacopoeial dissolution conditions (USP Apparatus 2 at 50 rpm with phosphate buffer (pH 5.8)) were tested [12]. An ultraviolet-visible (UV/Vis) spectrophotometer (Model Lambda 35, Perkin Elmer, USA) with 1 mm flow cells was used. The equipment was controlled by specific software designed by Sotax. Furosemide tablets were sprinkled on 900 mL of 0.1 N hydrochloric acid, acetate buffer (pH 4.5), and phosphate buffer (pH 6.8) at 37.0 ± 0.5 °C. Samples were taken automatically every 5 min for 60 min. Dissolved furosemide was quantified with standard calibration curves, in each dissolution medium, at 274 nm.

### Flow-through cell method

Dissolution profiles of furosemide were obtained with USP Apparatus 4 (Model CE6, Sotax AG, Basel, Switzerland) with 22.6 mm cells (i.d.). The laminar flow (originated with 6 g of glass beads) of 16 mL/min was tested. The dissolution media also comprised 0.1 M hydrochloric acid, acetate buffer (pH 4.5), and phosphate buffer (pH 6.8) at 37.0 ± 0.5 °C. Samples were taken automatically every 5 min for 60 min. Dissolved furosemide was quantified in a UV/Vis spectrophotometer (Model Lambda 10, Perkin Elmer, USA) with 1 mm cells at 274 nm. For every trial, and depending on the schedule work, a standard calibration curve in 0.1 N hydrochloric acid, acetate buffer (pH 4.5) or phosphate buffer (pH 6.8) was prepared.

### Dissolution data analysis

Dissolution profiles were compared with model-independent and model-dependent approaches. For model-independent comparisons, mean dissolution time (MDT) and dissolution efficiency (DE) were calculated. MDT is the time to dissolve 63.2% of the drug and was calculated according to the statistical moment’s theory [22, 23]. Other authors have indicated the MDT to be 62–64% [24]. DE is the area under the dissolution curve up to a certain time, t, expressed as a percentage of the area of the rectangle described by 100% dissolution in the same period [25]. Both parameters were calculated with the Excel add-in DDSolver program [26].

For model-dependent comparisons, dissolution data were adjusted to a hyperbole equation (y = ax/b+x) and, using a and b parameters, time to 50% dissolution (*t*_50%_) and time to 80% dissolution (*t*_80%_) were calculated. The fit was calculated using SigmaPlot software (version 11.0). For a complete comparison of dissolution data by a model-dependent approach, dissolution data were fitted to zero-order, first-order, Higuchi, Hixson-Crowell, Makoid-Banakar, and Weibull models. The model with the highest adjusted determination coefficient (*R*^2^_adjusted_) and lowest Akaike information criterion (AIC) is the best-fit model [27]. Data analysis was carried out using Excel add-in DDSolver program [28]. All statistical comparisons were carried out with Student’s t-tests with significant differences at p < 0.05.

## Results

### Content uniformity and assay

The drug product used met the content uniformity and assay tests specified in the USP. The percentage of furosemide in the content uniformity test ranged from 97.4–100.31% (pharmacopoeial criteria, 85–115%) and that in the assay test was 100.79% (criteria, 90–110%) [12].

### Dissolution profiles with USP basket and paddle apparatus

**Figure 2.**
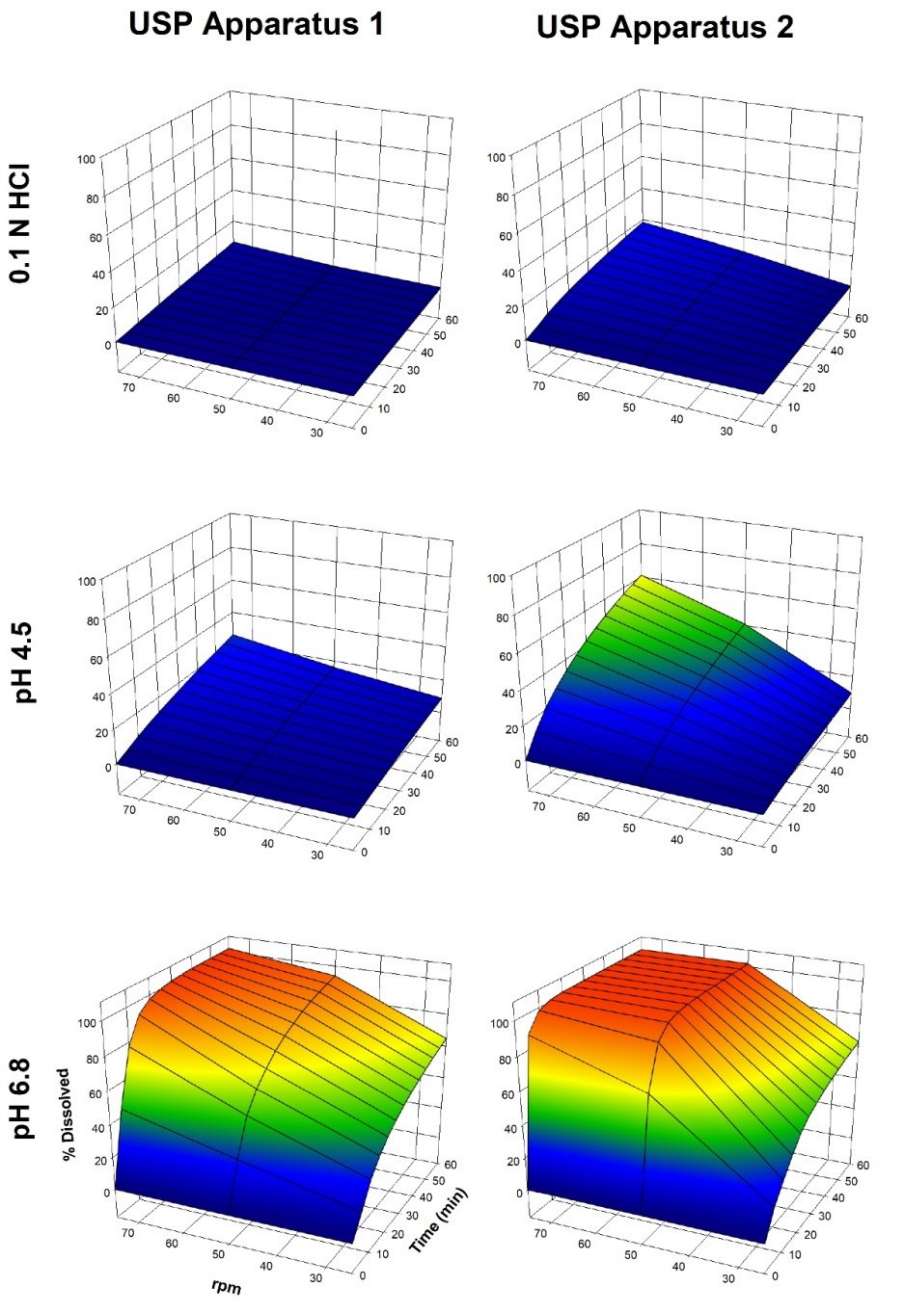
Dissolution profiles of furo¬semide reference tablets using USP Apparatuses 1 and 2 with dissolution media in physiological pH range. Mean, n = 6.

Dissolution profiles of furosemide obtained with the USP Apparatuses 1 and 2 at different agitation rates and in different dissolution media are shown in Figure 2.

A limited amount of furosemide dissolved in 0.1 M hydrochloric acid at 60 min with both USP dissolution apparatuses (< 20%). Almost 60% of the drug dissolved using USP Apparatus 2 at 75 rpm with acetate buffer (pH 4.5) as the dissolution medium. A complete release of the drug was achieved using phosphate buffer (pH 6.8) at 50 and 75 rpm, independent of the USP apparatus used. To compare dissolution profiles between USP Apparatuses 1 and 2, model-independent and -dependent parameters, at pH 6.8, were calculated, the results of which are shown in Table 1.

At 25 rpm and with both USP apparatuses, less than 65% of furosemide dissolved; thus, the *t*_80%_ was not calculated. At this agitation rate, significant differences in MDT values were observed (p < 0.05). At 50 rpm, significant differences in all calculated parameters were observed (p < 0.05). These results suggest a complete non-equivalence in the dissolution performance of furosemide between USP Apparatuses 1 and 2 at these conditions (pH 6.8 and 50 rpm). At 75 rpm, significant differences in MDT, DE, *t*_50%_, and *t*_80%_ values were observed (p < 0.05). Higher values of MDT, *t*_50%_, and *t*_80%_ were observed with USP Apparatus 1 than with USP Apparatus 2, at 50 and 75 rpm; these findings could be attributed to slower *in vitro* dissolution rates in USP Apparatus 1. Dissolution data at pH 6.8, adjusted with different mathematical models, are shown in Table 2.

Considering the established criteria to choose the best-fit model (higher *R*^2^_adjusted_ and lower AIC values), the Weibull function was the best mathematical equation to describe all dissolution data at pH 6.8. The expression of this function is as follows [26]:


(1)

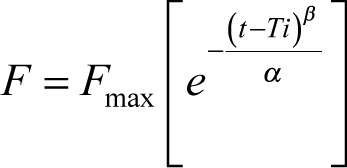



where *F* is the percent of the drug that dissolved *vs*. *t* time, *F*_max_ is the maximum percent of the drug that dissolved at infinite time, α is the scale factor of the process, *β* is the shape factor, and *T*i is a location parameter of time in which the drug begins to dissolve. The furosemide dissolution data of both USP apparatuses adjusted to the Weibull model dissolution profiles were statistically compared with Td values derived from fitting to this equation. The *T*_d_ value represents the time interval necessary to dissolve or release 63.2% of the drug present in the pharmaceutical dosage form [25] and coincides with MDT if the dissolution rate-time curve can be approximated by a monoexponential equation [22]. The mean values of *α*, *β*, *T*i, *F*_max_, and *T*_d_ are shown in Table 3. Significant differences were observed in all comparisons (p < 0.05).

Model-dependent comparisons (by comparing *T*_d_ values) indicated that USP Apparatuses 1 and 2 at 50 and 75 rpm generated different dissolution profiles. As both USP dissolution apparatuses and agitation rates created different hydrodynamic environments, these results were expected.

### Dissolution profiles with flow-through cell method

**Figure 3.**
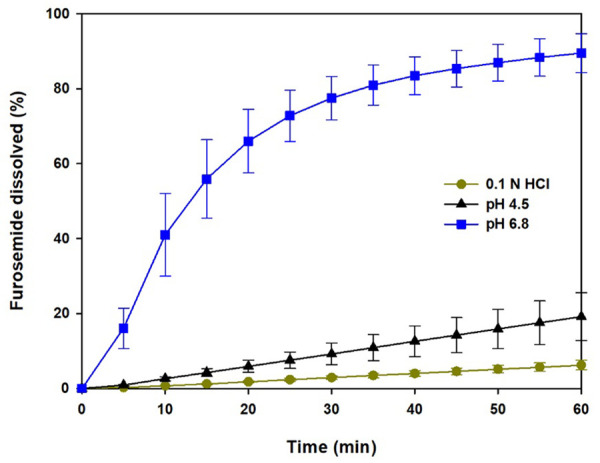
Dissolution profiles of furosemide reference tablets using USP Apparatus 4 with dissolution media in physiological pH range. Mean ± SD, n = 12.

Dissolution profiles of furosemide reference tablets using USP Apparatus 4 with 0.1 M hydrochloric acid, acetate buffer (pH 4.5), phosphate buffer (pH 6.8), and laminar flow of 16 mL/min are shown in Figure 3.

**Figure 4.**
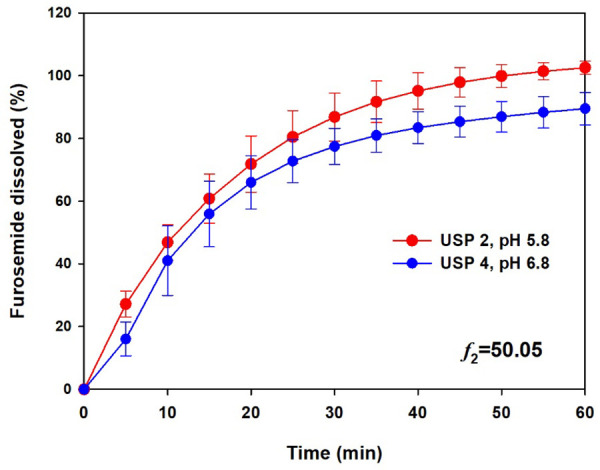
Dissolution profiles of furosemide reference tablets using the pharmacopoeial conditions (USP 2) and flow-through cell method (USP 4) with different dissolution media. Mean ± SD, n = 12.

With the flow-through cell method, less than 20% of furosemide dissolved when 0.1 M hydrochloric acid and acetate buffer (pH 4.5) were used, whereas almost 90% of the drug was released with phosphate buffer (pH 6.8). Slower dissolution rates with USP Apparatus 4 than with USP Apparatuses 1 and 2 have been found; however, in this case, a dissolution medium with low pH was an important factor for the low dissolution of furosemide. As more than 80% of the drug dissolved at 60 min at pH 6.8 and, for comparative purposes, the dissolution profile of furosemide tablets was obtained using a pharmacopoeial method. A test was carried out with USP Apparatus 2 at 50 rpm with 900 mL of phosphate buffer (pH 5.8) (Q = 80% at 60 min), the results of which are shown in Figure 4.

Under official conditions, the furosemide tablets met the Q pharmacopoeial specification (> 80% dissolved at 60 min). The dissolution profiles of USP Apparatuses 2 and 4 were similar (*f*_2_ > 50). This result suggested that a pharmacopoeial method (USP Apparatus 2) can produce a similar dissolution profile to that obtained with equipment (USP Apparatus 4) that generates a hydrodynamic environment similar to that inside the gastrointestinal tract and for which a correlation with *in vivo* data has been shown [19, 20]. For a complete comparison between the profiles, model-independent and -dependent parameters were calculated and statistically compared, the results of which are shown in Table 4.

Significant differences were observed in percent dissolved at 60 min, DE, and *t*_80%_ values (p < 0.05), whereas there was no difference in MDT and *t*_50%_, between USP Apparatuses 2 and 4. If we consider MDT and *t*_50%_ as parameters that reflect the *in vitro* dissolution rate, this is similar between the conditions (USP Apparatus 2 at 50 rpm and phosphate buffer (pH 5.8)/USP Apparatus 4 with a laminar flow of 16 mL/min and phosphate buffer (pH 6.8)), at least until the time at which 63.2% of the drug is dissolved. Furosemide dissolution data obtained with pharmacopoeial conditions and the flow-through cell method, adjusted to mathematical models, are shown in Table 5.

The Weibull function was the best-fit model to describe the *in vitro* release performance of furosemide reference tablets in these two dissolution apparatuses. Weibull parameters and Td values are shown in Table 6. No significant differences were observed in the Td data between the apparatuses (p > 0.05).

The dissolution of furosemide reference tablets using USP Apparatus 4 exceeded the Q pharmacopoeial criterion (only at pH 6.8) set for certain conditions in the USP Apparatus 2. When the dissolution data of USP Apparatus 4 were compared with those of USP Apparatus 2 (50 rpm and phosphate buffer pH 5.8), an equivalent *in vitro* release performance was achieved based on *f*_2_, MDT, *t*_50%_, and *T*_d_ comparisons.

Under all conditions used, furosemide dissolution profiles were well described by Weibull function. The shape factor of the Weibull function characterises the dissolution profile as exponential (*β* = 1); sigmoidal, with upward curvature followed by a turning point (β > 1); or parabolic, with steeper initial slope consistent with exponential (*β* < 1) [28]. In this case, furosemide tablets evaluated with USP Apparatus 1 at 75 rpm and pH 6.8, as well as with USP Apparatus 2 at 50 rpm and pH 5.8, generated *β* values > 1, meaning sigmoidal profiles.

## Discussion

Several authors have studied the effect of the hydrodynamic environment on the tablet dissolution rate. Wu *et al*. [29] studied the rate process that underlies tablet dissolution to understand the role of external hydrodynamics on mass transfer rate and film thickness during dissolution. Shah *et al*. [30] stated that the proper medium and appropriate rotational speed of the basket and paddle are of great importance to assure that the test procedure used is useful and discriminatory. Additionally, Levy *et al*. [31] concluded that the *in vitro* dissolution rate correlates with the *in vivo* absorption rate only at a low agitation rate (55 rpm). These results support the search for better dissolution schemes, especially with drugs with poor solubility. Poor permeability is a problem in the design of solid oral dosage forms with good bioavailability; thus, it is important to understand the influence of agitation rate, USP apparatus, and dissolution media on the *in vitro* release performance of class IV drugs.

The search for adequate dissolution conditions is not limited to poorly soluble drugs or the use of USP Apparatuses 1 and 2. Shabir [32] indicated that the rate of *in vitro* release of a hydrosoluble drug can be accurately controlled through the USP apparatus. Shabir worked with atenolol (class III drug) generic tablets using USP basket and paddle apparatuses. Although these dissolution apparatuses are currently the most popular methods, Gao [33] explains that both methods are operated under closed finite sink conditions and cannot mimic the conditions in the gastrointestinal tract. The flow-through cell method has gained recent acceptance into the dissolution field for its versatility in the testing of novel dosage forms where traditional dissolution apparatuses and methods have failed [34].

USP Apparatus 4 has several advantages: 1. sink conditions can be maintained for poorly soluble drugs throughout the dissolution run; 2. it is easy to change media (suggested media is physiological pH range) and modify flow rate to simulate *in vivo* conditions; 3. it simulates intraluminal hydrodynamics efficiently; 4. it can be modified for different dosage forms; and 5. it measures the *in vitro* release rate profile as an output that is similar to the shape of an *in vivo* profile [35]. American and European pharmacopoeias suggest three flow rates for testing with USP Apparatus 4 (4, 8, and 16 mL/min) [36]. In this *in vitro* release study of furosemide reference tablets, we used only the flow rate of 16 mL/min as the Sotax equipment model CE6 has a working flow range of 10‒50 mL/min.

Based on these characteristics, it is possible to establish a meaningful IVIVC with USP Apparatus 4. When a meaningful IVIVC has been established, it can be used as a surrogate for and to minimize the number of bioequivalence studies during drug product development [37]. Some authors have reported a better estimate of the absorption rate of cilostazol [19] and diclofenac sodium [20] formulations, both drugs with solubility problems, with the flow-through cell method.

Some drugs can be dissolved using dissolution media in the physiological pH range (pH 1.2, 4.5, and 6.8) or biorelevant media, such as FaSSIF and FeSSIF (media that simulate the absence or presence of food, respectively) [38], to document the release performance of oral dosage forms through the gastrointestinal tract. This is especially important with poorly soluble drugs, such as furosemide. Based on the physicochemical characteristics of this drug, dissolution at low pH is not physiologically relevant. For a complete dissolution scheme in a physiological pH range, the Food and Drug Administration recommends dissolution tests with a 0.1 N hydrochloric acid or simulated gastric fluid USP without enzymes, pH 4.5 or pH 6.8 buffer, or simulated intestinal fluid USP without enzymes [39]. The dissolution of furosemide reference tablets was carried out under these conditions, with the exception of simulated fluids, and the best results were observed with phosphate buffers at pH 5.8 and pH 6.8. More than 80% of the drug dissolved at 60 min and time parameters, such as MDT, *t*_50%_, and *t*_80%_ were calculated. At 50 rpm, significant differences in all calculated parameters (USP Apparatus 1 *vs*. 2) were observed. Differences between both USP apparatuses were expected owing to the different hydrodynamics of each apparatus; however, it is necessary to understand at what agitation rate this difference is more evident. Despite the wide use of USP Apparatuses 1 and 2 at different agitation rates, they have still been evaluated by several authors in terms of the surrounding hydrodynamic environments, which do not adequately reproduce the natural environment of the gastrointestinal tract [13-15, 32, 40].

The fitting of dissolution data to mathematical models was carried out without any physiological significance to discover the best equation to explain the *in vitro* release performance of furosemide reference tablets. These models were used to facilitate the analysis and interpretation of dissolution data because they describe the dissolution profiles as a function of the few parameters that can be statistically compared [41]. Han *et al*. [42] documented the first-order kinetics as the best-fit model to adjust furosemide dissolution data from commercial tablets; however, an incomplete fit scheme was created as only zero-order and first-order kinetics were used to adjust their dissolution data. In our study, after testing several equations (including common dissolution kinetics), Weibull function was the best mathematical model to explain the dissolution performance of furosemide.

The development of more discriminative methods than using pharmacopoeial conditions to evaluate the biopharmaceutical quality of generic formulations has been documented. Studies with class II drugs, such as carbamazepine [43], meloxicam [44], and naproxen sodium [45], tested with USP Apparatuses 2 and 4 have shown that USP Apparatus 2 may not reflect the dissolution performance of generic formulations and references. The choice of the hydrodynamic environment for the drug release is key to identify a meaningful IVIVC [35]. For class II drugs, IVIVCs have been identified and, with *in vitro* studies, they provide a good estimate of the absorption rate of class II drugs. Several authors have shown this important association only with USP Apparatus 4 [19, 20, 35]. However, similar dissolution profiles for USP Apparatuses 2 and 4 have been reported for naproxen sodium tablets [45] and ibuprofen suspensions [46]. These results are important where no flow-through cell method is available, and an equivalent hydrodynamic environment is required to test solid dosage forms.

## Conclusions

The *in vitro* release performance of furosemide reference tablets was determined using USP Apparatuses 1 and 2 at different agitation rates and dissolution media of physiological relevance. A limited amount of furosemide dissolved with both at pH 1.2 and 4.5. Better results were obtained with a dissolution medium of pH 6.8. Although USP basket and paddle apparatuses are the most widely used, it is important to take advantage of USP Apparatus 4 for the evaluation of furosemide tablets under the hydrodynamic environment that this equipment generates. All information collected is important to reduce the negative clinical impact that this class IV drug presents. This furosemide reference product is the comparative formulation for generic drug products; thus, it is important to understand the *in vitro* release performance under all possible schemes for the design of better commercial formulations.

## Figures and Tables

**Table 1.** Model-independent and -dependent parameters of furosemide at pH 6.8. Mean ± SEM, n = 6.

Agitation rate (rpm)	Parameter	USP Apparatus 1	USP Apparatus 2
25	Diss. at 60 min (%)	64.61 ± 1.64	62.98 ± 5.87
	MDT (min)	18.94 ± 0.16	17.11 ± 0.42*
	DE (%)	44.42 ± 1.18	45.15 ± 4.49
	*t*_50%_ (min)	31.09 ± 1.89	33.07 ± 8.47
	*t*_80%_ (min)	†	†
50	Diss. at 60 min (%)	93.90 ± 2.23	101.76 ± 0.54*
	MDT (min)	15.00 ± 0.81	4.99 ± 0.14*
	DE (%)	70.56 ± 2.85	93.30 ± 0.43*
	*t*_50%_ (min)	11.15 ± 1.35	2.07 ± 0.11*
	*t*_80%_ (min)	32.27 ± 4.02	6.75 ± 0.30*
75	Diss. at 60 min (%)	102.43 ± 0.30	102.02 ± 0.48
	MDT (min)	7.49 ± 0.41	3.42 ± 0.01*
	DE (%)	89.64 ± 0.77	96.20 ± 0.45*
	*t*_50%_ (min)	4.13 ± 0.30	0.72 ± 0.02*
	*t*_80%_ (min)	11.95 ± 0.75	2.58 ± 0.08*

*p < 0.05.

†Data not calculated

**Table 2.** Criteria used for the selection of the best-fit model at pH 6.8. Mean, n = 6.

Parameter	Agitation rate (rpm)	Zero-order	First-order	Higuchi	Hixson-Crowell	Makoid-Banakar	Weibull
USP Apparatus 1							
*R* ^2^_adjusted_	25	0.5474	0.8935	0.9731	0.8155	0.9938	0.9997
	50	-0.2118	0.9725	0.8774	0.8929	0.9932	0.9996
	75	-3.99	0.9492	-0.2872	0.6144	0.9281	0.9994
AIC	25	87.76	69.95	53.49	76.81	37.07	1.85
	50	104.11	56.38	74.14	74.83	42.38	8.99
	75	117.66	60.73	101.10	85.25	66.30	0.64
USP Apparatus 2							
*R* ^2^_adjusted_	25	0.3232	0.7889	0.9521	0.6872	0.9952	0.9976
	50	-17.85	0.9611	0.8774	-1.85	0.9932	0.9999
	75	-144.29	0.7176	-50.97	-31.37	0.9016	0.9998
AIC	25	90.15	73.14	55.70	79.54	27.37	17.52
	50	120.96	36.84	74.14	98.05	42.38	-34.36
	75	123.27	42.04	110.92	105.23	37.35	-40.46

**Table 3.** Weibull parameters and Td values at pH 6.8. Mean, n = 6.

Agitation rate (rpm)	*α*	*β*	*T*i	*F*_max_	*T*_d_ ± SEM (min)
USP Apparatus 1					
25	10.25	0.63	3.12	95.48	50.15 ± 9.38
50	5.81	0.59	2.70	111.03	22.78 ± 3.93
75	13.30	1.15	0.72	102.43	7.64 ± 0.43
USP Apparatus 2					
25	30.04	0.94	-0.11	70.50	25.63 ± 3.51*
50	2.75	0.84	1.53	101.76	4.72 ± 0.18*
75	1.87	0.77	-0.39	102.03	1.80 ± 0.12*

*p < 0.05.

**Table 4.** Model-independent and -dependent parameters of furosemide. Mean ± SEM, n = 12.

Parameter	USP Apparatus 2 (pH 5.8)	USP Apparatus 4 (pH 6.8)
Diss. at 60 min (%)	102.55 ± 0.61	89.49 ± 1.50*
MDT (min)	15.61 ± 0.68	15.28 ± 1.06
DE (%)	75.93 ± 1.52	66.57 ± 1.44*
*t*_50%_ (min)	10.86 ± 0.79	13.35 ± 1.10
*t*_80%_ (min)	25.99 ± 1.50	36.64 ± 2.34*

*p < 0.05.

**Table 5.** Criteria used for the selection of the best-fit model. Mean, n = 12.

Parameter	USP Apparatus	Zero-order	First-order	Higuchi	Hixson-Crowell	Makoid-Banakar	Weibull
R^2^_adjusted_	2 (pH 5.8)	0.1571	0.9788	0.9152	0.9882	0.9770	0.9994
	4 (pH 6.8)	0.0115	0.9205	0.8048	0.8488	0.9743	0.9991
AIC	2 (pH 5.8)	104.08	58.28	75.30	52.62	30.04	8.29
	4 (pH 6.8)	101.87	66.11	81.14	74.67	57.47	15.79

**Table 6.** Weibull parameters and Td values. Mean, n = 12.

USP Apparatus	*α*	*β*	*T*i	*F*_max_	*T*_d_ ± SEM (min)
2 (pH 5.8)	626.71	1.13	-1.99	110.39	20.89 ± 3.93
4 (pH 6.8)	12.22	0.89	3.12	92.57	16.63 ± 1.43

*p < 0.05

## References

[1] Abou-Auda HS Al-Yamani MJ Morad AM Bawazir SA Khan SZ Al-Khamis KI. High-performance liquid chromatographic determination of furosemide in plasma and urine and its use in bioavailability studies. J Chromatogr B Biomed Sci Appl. 710 (1998) 121–8. 9686878 10.1016/s0378-4347(98)00058-9

[2] Berthod A. Hydrophobicity of ionizable compounds. A theoretical study and measurements of diuretic octanol-water partition coefficients by countercurrent chromatography. Anal Chem. 71 (1999) 879–88.

[3] Granero GE Longhi MR Mora MJ Junginger HE Midha KK Shah VP Biowaiver monographs for immediate-release solid oral dosage forms: furosemide. J Pharm Sci. 99 (2010) 2544–56. 19960529 10.1002/jps.22030

[4] Tambosi G Coelho PF Soares L Schmücker Lenschow IC Zétola M Stulzer HK Challenges to improve the biopharmaceutical properties of poorly water-soluble drugs and the application of the solid dispersion technology. Revista Matéria. 23 (2018) e-12224.

[5] García-Arieta A. Interactions between active pharmaceutical ingredients and excipients affecting bioavailability: impact on bioequivalence. Eur J Pharm Sci. 65 (2014) 89–97. 25236823 10.1016/j.ejps.2014.09.004

[6] Ghadi R Dand N. BCS class IV drugs: highly notorious candidates for formulation development. J Control Release. 248 (2017) 71–95. 28088572 10.1016/j.jconrel.2017.01.014

[7] Kelly M.R. Cutler R.E. Forrey A.W. Kimpel B.M.. Pharmacokinetics of orally administered furosemide. Clinical Pharmacology and Therapeutics 15 (1973) 178˗186. 4812154

[8] Sweetman S. Martindale: The complete drug reference. (Electronic version) Pharmaceutical Press. Thomson/MICROMEDEX, London, UK/Greenwood Village, Colorado. 2009.

[9] Grahnén A. Hammarlund M. Lundqvist T.. Implications of intraindividual variability in bioavailability studies of furosemide. European Journal of Clinical Pharmacology 27 (1984) 595˗602. 6519165 10.1007/BF00556898

[10] Kaojarern S. Poobrasert O. Utiswannakul A. Kositchaiwat U.. Bioavailability and pharmacokinetics of furosemide marketed in Thailand. Journal of the Medical Association of Thailand 73 (1990) 191˗197. 2394955

[11] Stüber W Mutschler E Steinbach D. The pharmaceutical and biological availability of commercial preparations of furosemide. Arzneimittelforschung Drug Research. 32 (1982) 693–7. 6889429

[12] United States Pharmacopeia and National Formulary USP 42-NF 37; United States Pharmacopeial Convention, Inc: Rockville MD; 2019.

[13] Morihara M Aoyagi N Kaniwa N Katori N Kojim S. Hydrodynamic flows around tablets in different pharmacopeial dissolution tests. Drug Dev Ind Pharm. 28 (2002) 655–62. 12149957 10.1081/ddc-120003856

[14] Greco K Bergman TL Bogner R. Design and characterization of laminar flow-through dissolution apparatus: comparison of hydrodynamic conditions to those of common dissolution techniques. Pharm Dev Technol. 16 (2011) 75–87. 20105086 10.3109/10837450903499341

[15] Todaro V Persoons T Grove G Healy MA D’Arcy DM. Characterization and simulation of hydrodynamics in the paddle, basket and flow-through dissolution testing apparatuses - a review. Dissolut Technol. 24 (2017) 24–36.

[16] Bhattachar SN Wesley JA Fioritto A Martin PJ Babu SR. Dissolution testing of a poorly soluble compound using the flow-through cell dissolution apparatus. Int J Pharm. 236 (2002) 135–43. 11891077 10.1016/s0378-5173(02)00027-3

[17] Beyssac E Lavigne J. Dissolution study of active pharmaceutical ingredients using the flow through apparatus USP 4. Dissolut Technol. 12 (2005) 23–5.

[18] Fotaki N Reppas C. The flow through cell methodology in the evaluation of intralumenal drug release characteristics. Dissolut Technol. 12 (2005) 17–21.

[19] Jinno J Kamada N Miyake M Yamada K Mukai T Odomi M In vitro-in vivo correlation for wet-milled tablet of poorly water-soluble cilostazol. J Control Release. 130 (2008) 29–37. 18582979 10.1016/j.jconrel.2008.05.013

[20] Jantratid E De Maio V Ronda E Mattavelli V Vertzoni M Dressman JB. Application of biorelevant dissolution tests to the prediction of *in vivo* performance of diclofenac sodium from an oral modified-release pellet dosage form. Eur J Pharm Sci. 37 (2009) 434–41. 19491035 10.1016/j.ejps.2009.03.015

[21] Listado Actualizado de medicamentos de referencia 2019/01, https://www.gob.mx/cms/uploads/attachment/file/441323/Listado_de_medicamentos_de_Referencia_.pdf, accessed on 04 March 2020.

[22] Tanigawara Y Yamaoka K Nakagawa T Unzo T. New method for the evaluation of *in vitro* dissolution time and disintegration time. Chem Pharm Bull (Tokyo). 30 (1982) 1088–90.

[23] Collier PS. The interpretation of in vivo mean dissolution time data. In: Benet L.Z. Levy G. Ferraiolo B.L. (eds) Pharmacokinetics. Springer-Verlang. Boston, USA. 1984.

[24] Podczeck F. Comparison of in vitro dissolution profiles by calculating mean dissolution time (MDT) or mean residence time (MRT). Int J Pharm. 97 (1993) 93–100.

[25] Costa P Sousa Lobo JM. Modeling and comparison of dissolution profiles. Eur J Pharm Sci. 13 (2001) 123–33. 11297896 10.1016/s0928-0987(01)00095-1

[26] Zhang Y. Huo M. Zhou J. Zou A. Li W. Yao C. Xie S.. DD Solver: an add-in program for modeling and comparison of drug dissolution profiles. The AAPS Journal 12 (2010) 263˗271. 20373062 10.1208/s12248-010-9185-1 PMC2895453

[27] Yuksel N Kanik AE Baykara T. Comparison of *in vitro* dissolution profiles by ANOVA-based, model-dependent and independent-methods. Int J Pharm. 209 (2000) 57–67. 11084246 10.1016/s0378-5173(00)00554-8

[28] Langenbucher F. Linearization of dissolution curves by the Weibull distribution. J Pharm Pharmacol. 24 (1972) 979–81. 4146531 10.1111/j.2042-7158.1972.tb08930.x

[29] Wu Y Kildsing DO Ghaly ES. Effect of hydrodynamic environment on tablet dissolution rate. Pharm Dev Technol. 9 (2004) 25–37. 15000464 10.1081/pdt-120027415

[30] Shah VP Gurbarg M Noory A Dighe S Skelly JP. Influence of higher rates of agitation on release patterns of immediate-release drug products. J Pharm Sci. 81 (1992) 500–3. 1522485 10.1002/jps.2600810604

[31] Levy G Hayes BA. Physicochemical basis of the buffered acetylsalicylic acid controversy. N Engl J Med. 262 (1960) 1053–8. 14416389 10.1056/NEJM196005262622102

[32] Shabir GA. Evaluation of USP basket and paddle dissolution methods using different generic atenolol tablets. Turkish Journal of Pharmaceutical Sciences. 8 (2011) 253–60.

[33] Gao Z. *In vitro* dissolution testing with flow-through method: a technical note. AAPS PharmSciTech. 10 (2009) 1401–5. 19937283 10.1208/s12249-009-9339-6 PMC2799605

[34] Singh I Aboul-Eneim HY. Advantages of USP Apparatus IV (flow-through cell apparatus) in dissolution studies. J Indian Chem Soc. 3 (2006) 220–2.

[35] Fang JB Robertson VK Rawat A Flick T Tang ZJ Cauchon NS Development and application of a biorelevant dissolution method using USP Apparatus 4 in early phase formulation development. Mol Pharm. 7 (2010) 1466–77. 20701327 10.1021/mp100125b

[36] Steffansen B Brodin B Nielsen C. Molecular biopharmaceutics: aspects of drug characterization, drug delivery and dosage form evaluation. ULLA Pharmacy Series. Pharmaceutical Press. London, United Kingdom. 2009.

[37] Larsen C Larsen SW Jensen H Yaghmur A Østergaard J. Role of *in vitro* release models in formulation development and quality control of parenteral depots. Expert Opin Drug Deliv. 6 (2009) 1283–95. 19941410 10.1517/17425240903307431

[38] Fagerberg JH Tsinman O Sun N Tsinman K Avdeef A Bergström CAS. Dissolution rate and apparent solubility of poorly soluble drugs in biorelevant dissolution media. Mol Pharm. 7 (2010) 1419–30. 20507160 10.1021/mp100049m

[39] US DHHS. FDA, CDER, Guidance for Industry: Waiver if in vivo bioavailability and bioequivalence studies for immediate-release solid oral dosage forms based on a Biopharmaceutics Classification System, U.S. Department of Health and Human Services, Food and Drug Administration, Center for Drug Evaluation and Research (CDER) (2017), Rockville, MD, USA.

[40] Missaghi S Fassihi R. Release characterization of dimenhydrinate from an eroding and swelling matrix: selection of appropriate dissolution apparatus. Int J Pharm. 293 (2005) 35–42. 15778042 10.1016/j.ijpharm.2004.12.024

[41] Adams E Coomans D Smeyers-Verbeke J Massart DL. Non-linear mixed effects models for the evaluation of dissolution profiles. Int J Pharm. 240 (2002) 37–53. 12062500 10.1016/s0378-5173(02)00127-8

[42] Han YK Simionato LD Calvo RG Mattei MB Segall AI. Comparison of dissolution profiles of furosemide tablets available in the Argentinian market. Journal of Applied Solution Chemistry and Modeling. 3 (2014) 186–93.

[43] Medina JR Salazar DK Hurtado M Cortés AR Domínguez-Ramírez AM. Comparative *in vitro* dissolution study of carbamazepine immediate-release products using the USP paddles method and the flow-through cell system. Saudi Pharm J. 22 (2014) 141–7. 24648826 10.1016/j.jsps.2013.02.001 PMC3950529

[44] Medina-López JR Orozco-Juárez JA Hurtado M. Dissolution performance of meloxicam formulations under hydrodynamics of USP paddle apparatus and flow-through cell method. International Journal of Applied Pharmaceutics. 11 (2019) 182–8.

[45] Medina JR Uribe A Hurtado M Domínguez-Ramírez AM. *In vitro* equivalence study of generic naproxen tablets using the USP paddle apparatus and the flow-through cell method. Int J Pharm Pharm Sci. 7 (2015) 348–54.

[46] Medina JR Cortes M Romo E. Comparison of the USP Apparatus 2 and 4 for testing the *in vitro* release performance of ibuprofen generic suspension. International Journal of Applied Pharmaceutics. 9 (2017) 90–5.

